# Deciphering mechanisms of acquired T790M mutation after EGFR inhibitors for NSCLC by computational simulations

**DOI:** 10.1038/s41598-017-06632-y

**Published:** 2017-07-26

**Authors:** Bin Zou, Victor H. F. Lee, Lijiang Chen, Lichun Ma, Debby D. Wang, Hong Yan

**Affiliations:** 1 Department of Electronic Engineering, City University of Hong Kong, Kowloon, Hong Kong China; 2 Department of Clinical Oncology, Li Ka Shing Faculty of Medicine, The University of Hong Kong, Pokfulam, Hong Kong China; 3 School of Electronic and Information Engineering, Beihang University, Beijing, China; 4 School of Computing and Information Sciences, Caritas Institute of Higher Education, Hong Kong, China

## Abstract

Metastatic non-small-cell lung cancer (NSCLC) with activating EGFR mutations responds very well to first and second generation tyrosine-kinase inhibitors (TKI) including gefitinib, erlotinib and afatinib. Unfortunately, drug resistance will eventually develop and about half of the cases are secondary to the emergence of acquired T790M somatic mutation. In this work, we prospectively recruited 68 patients with metastatic EGFR-mutated NSCLC who have developed progressive disease after first-line TKI with or without subsequent TKI and/or other systemic therapy. Liquid biopsy after progression to their last line of systemic therapy were taken for detection of acquired T790M mutation. By performing attribute ranking we found that several attributes, including the initial EGFR mutational type, had a high correlation with the presence of acquired T790M mutation. We also conducted computational studies and discovered that the EGFR mutation delE746_A750 had a lower stability around the residue T790 than delS752_I759 and L858R, which was consistent with our clinical observation that patients with delE746_A750 were more likely to acquire T790M mutation than those with delS752_I759 or L858R. Our results provided new insight to future direction of research on investigating the mechanisms of acquired T790M mutation, which is essential to the development of novel mutation-specific TKIs.

## Introduction

Lung cancer is the leading cause of cancer death worldwide^1, 2^. Non-small-cell lung cancer (NSCLC) constitutes about 85% of all lung cancers with adenocarcinoma as the most common histological type. Mutation of the epidermal growth factor receptor (EGFR) is the most common driver mutation of NSCLC and its incidence can reach 60% in East Asian population^3–5^. These “driver” mutations increase the kinase activity of EGFR, leading to EGFR overexpression and uncontrolled lung cell division and eventually lung cancer. Exon 19 deletions and exon 21 L858R point mutation account for more than 80% of driver mutations^6, 7^.

Tyrosine-kinase inhibitors (TKI) against EGFR-mutated NSCLC have been devised and investigated extensively for the past 2 decades. First-generation TKI including gefitinib and erlotinib, are reversible inhibitors binding to the EGFR kinase domain, which block the subsequent signal transduction leading to inhibition of tumor proliferation. International phase III randomized-controlled trials (RCT) have confirmed their superiority with improved progression-free survival (PFS) over systemic chemotherapy as first-line treatment for metastatic EGFR-mutated NSCLC^8–15^. Afatinib, as a second-generation TKI which binds irreversibly to ErbB family receptors, has also been shown to confer PFS advantage over systemic chemotherapy, and more recently, gefitinib as first-line treatment^16–18^. Tumors with activating EGFR mutations, especially exon 19 deletion, are particularly responsive to this second-generation TKI leading to a longer overall survival (OS) compared to systemic chemotherapy^19^. Based on these outcomes with improved PFS, better objective responses and more manageable toxicity profiles over systemic chemotherapy, these three TKIs have been approved by Food and Drug Administration of the United States as first-line treatment for metastatic EGFR-mutated NSCLC. Despite initial promising and dramatic response to these TKIs, almost drug resistance will ultimately develop within 1 to 2 years. The most common mechanism of drug resistance is the development of somatic mutation T790M on exon 20, accounting for about 50% of all mutations of acquired resistance^20^.

There are two plausible explanations for the development of T790M mutation as acquired resistance to EGFR TKI therapy. As threonine 790 is located at the entrance in the back of the ATP binding cleft, one postulation is that substitution of threonine 790 with a bulky methionine causes steric interference with binding of TKIs^20–22^. Another explanation is that introduction of the T790M mutation increases the affinity for adenosine triphosphate (ATP) which in turn causes reduced binding of the ATP-competitive TKI including gefitinib and erlotinib^20, 21, 23^. Nevertheless, little attention has been paid to the mechanism of emergence of T790M mutation. Very limited information is known hitherto with respect to the reason why T790M emerges and the predictive factors for such development.

In this study, we analyzed a prospectively collected cohort of patients with metastatic EGFR-mutated NSCLC treated with gefitinib, erlotinib or afatinib as first-line treatment with or without subsequent TKI or system chemotherapy. Plasma biopsy with or without additional paired tumor biopsies were performed at the time of progressive disease (PD) after their last line of systemic therapy for detecting acquired T790M mutation. We evaluated the correlation of each personal attribute and presence of T790M after one or more line(s) of TKIs therapy with or without additional systemic therapy. Moreover, computational modeling and molecular dynamics (MD) simulations were employed to investigate the involved EGFR mutants and their motion patterns when binding with these three TKIs. We then further analyzed the stability of residues around T790 for each mutant-TKI complex.

## Results

### Patient characteristics and therapy profiles

This study prospectively recruited 68 patients with EGFR-mutated NSCLC and their baseline characteristics were shown in Table 1. Almost all patients (98.5%) suffered from adenocarcinoma with the exception of one patient who suffered from squamous cell carcinoma. Baseline EGFR mutations were of the following types: exon 19 LRE deletions (most of them were delE746_A750, deletion of residues at sites 746–750) in 34 (50.0%) patients, exon 19 non-LRE deletions (deletion-starting codon other than L747, R748 nor E749) in 3 (4.4%) patients and exon 21 L858R point mutation in 31 (45.6%) patients. These three subgroups have been found prognostic of progression-free survival after first-line TKI therapy in our previous study^24^. Of the 34 patients with exon 19 LRE deletions, 31 of them had delE746_A750, while the remaining 3 patients had delE746_S752insV, delL747_T751insP and delE746_T751insV, respectively. The 3 patients with exon 19 non-LRE deletions had delS752_I759, delT751_I759insN and delT751_I759insS, respectively. Interestingly, 3 (4.4%) patients had double mutations, coupled with either exon 19 deletion (1 patient) or L858R point mutation (2 patients).

**Table 1:** Baseline patient characteristics.

	N = 68 (%)
Median age in years (range)	66 (47–87)
Sex	
Male	14 (20.6)
Female	54 (79.4)
Histology	
Adenocarcinoma	67 (98.5)
Squamous cell carcinoma	1 (1.5)
Smoking history	
Never smoker	58 (85.3)
Chronic smoker	2 (2.9)
Ex-smoker	8 (11.8)
ECOG PS	
0	20 (29.4)
1	48 (70.6)
Site of metastases before TKI therapy	
Lung	59 (86.8)
Pleura/pleural effusion	30 (44.1)
Brain	15 (22.1)
Liver	11 (16.2)
Bone	34 (50.0)
Distant lymph nodes	18 (26.5)
Number of sites of metastases before 1^st^ line TKI therapy	
1	15 (22.1)
2	12 (32.4)
3	19 (27.9)
4	9 (13.2)
5	3 (4.4)
Types of pre-treatment EGFR mutation	
Exon 19 mutations	37 (54.4)
LRE deletions	34 (50.0)
E746_A750 deletions	31 (45.6)
Non-E746_A750 deletions	3 (4.4)
Non-LRE deletions	3 (4.4)
Exon 21 L858R mutations	31 (45.6)
Double mutations	3 (4.4)
Exon 19 deletion + exon 20 D807E mutation	1 (1.5)
Exon 21 L858R mutation + exon V765L mutation	1 (1.5)
Exon 21 L858R mutation + exon 18 G719A mutation	1 (1.5)
Number of lines of TKI therapies before liquid biopsy	
1 TKI	53 (77.9)
2 TKIs	12 (17.6)
3 TKIs	3 (4.4)
First-line TKI used	68 (100)
Gefitinib	54 (79.4)
Erlotinib	7 (10.3)
Afatinib	7 (10.3)
Second-line TKI used	14 (20.6)
Gefitinib	2 (2.9)
Erlotinib	8 (11.8)
Afatinib	4 (5.9)
Third-line TKI used	3 (4.4)
Gefitinib	0 (0)
Erlotinib	2 (2.9)
Afatinib	1 (1.5)
Best overall response after 1^st^ (n = 68)/2^nd^ (n = 14)/3^rd^ (n = 3) TKI therapy before liquid biopsy	
Complete response	5 (7.4)/0 (0)/0 (0)
Partial response	39 (57.4)/2 (2.9)/0 (0)
Stable disease	21 (30.9)/6 (8.8)/2 (2.9)
Progressive disease	3 (4.4)/6 (8.8)/1 (1.5)
Number of lines of prior chemotherapy ± anti-VEGF therapy before liquid biopsy	
0 line	44 (64.7)
1 line	16 (23.5)
2 lines	6 (8.8)
3 lines	2 (2.9)
Best overall response after 1^st^ (n = 23)/2^nd^ (n = 9)/3^rd^ (n = 2) line chemotherapy ± anti-VEGF therapy before liquid biopsy	
Complete response	1 (1.5)/0 (0)/0 (0)
Partial response	7 (10.3)/4 (5.9)/(1 (1.5)
Stable disease	7 (10.3)/3 (4.4)/0 (0)
Progressive disease	8 (11.8)/2 (2.9)/1 (1.5)

ECOG: Eastern Cooperative Oncology Group; EGFR: epidermal growth factor receptor; PD: progressive disease; PS: performance status; TKI: tyrosine-kinase inhibitors; VEGF: vascular endothelial growth factor.

All patients received first-line TKI with or without additional TKI and/or systemic therapies. Before plasma and/or tumor re-biopsy for acquired T790M mutation, 51 (75%) patients received only one line of TKI, 14 (21%) patients received two lines of TKI and 3 (4%) patients received three lines of TKI. 16 (23.5%), 6 (8.8%) and 2 (2.9%) patients received one, two and three additional lines of chemotherapy with or without anti-vascular endothelial growth factor (anti-VEGF) therapy before plasma and/or tumor re-biopsy for acquired T790M mutation. All patients had plasma re-biopsy by ddPCR at the time of PD after their last line of systemic therapy. Four (5.9%) patients had additional paired tumor re-biopsies for acquired T790M mutation and their tumor genotyping results were all concordant with the plasma re-biopsy result.

### Attribute ranking results

To evaluate the importance of each attribute contributing to the presence of T790M mutation in tumor or plasma biopsy, we performed attribute ranking using six attribute ranking methods available in Weka 3.8.0. In Table 2, the first three selected attributes were listed for each attribute evaluator. We found that three attributes namely Bone_met, Number_sites_met and Initial_EGFR_mutation had the highest ranking for most attribute evaluators.

**Table 2:** Attribute ranking results.

	Attribute evaluator	Top three attributes
1	Chi-squared Ranking Filter	1. Bone_met
		2. Number_sites_met
		3. Initial_EGFR_mutation
2	Correlation Ranking Filter	1. Bone_met
		2. Initial_EGFR_mutation
		3. Age_60
3	Filtered Attribute Evaluator	1. Bone_met
		2. Number_sites_met
		3. Initial_EGFR_mutation
4	Information Gain Ranking Filter	1. Bone_met
		2. Number_sites_met
		3. Initial_EGFR_mutation
5	ReliefF Ranking Filter	1. Bone_met
		2. Initial_EGFR_mutation
		3. Age_75
6	Symmetrical Uncertainty Ranking Filter	1. Bone_met
		2. Number_sites_met
		3. Initial_EGFR_mutation

Altogether 27 (39.7%) patients acquired T790M mutation confirmed by plasma or tumor re-biopsy after TKI with or without subsequent TKI and/or systemic therapies. Also 34 (50.0%) patients had bone metastasis at baseline and 21 (61.8%) of them acquired T790M mutation after TKI therapy, accounting for 77.8% of all of the 27 patients who acquired T790M mutation. We observed that patients with bone metastasis at baseline were more likely to acquire T790M (61.8%) than patients without it (17.7%) (p = 0.0004). Moreover, 15 out of 18 (83.3%) patients who had both bone metastasis and delE746_A750 mutation at baseline developed acquired T790M mutation. If patients had both bone metastasis and delE746_A750 mutation, the probability of acquiring T790M was even higher (83.3%) (p < 0.0001). With respect to the number of sites of metastasis at baseline, 15 patients had only one site of metastasis and 14 (93.3%) of them did not acquire T790M. It was noted that if patients had only one site of metastasis, they were unlikely to develop T790M (p < 0.0001).

As far as the initial EGFR mutational types were concerned, 61.3% of patients with the delE746_A750 mutation acquired T790M, compared to no patients and 25.8% of patients with exon 19 non-LRE deletions and L858R mutation respectively (p = 0.0059). Table 3 showed the probability of acquiring T790M for each mutation and for each TKI. Interestingly, the other 3 patients with exon 19 LRE deletions but not delE746_A750 (not shown in Table 3) did not acquire T790M.

**Table 3:** Probability of acquiring T790M for each mutation and tyrosine-kinase inhibitor.

	delE746_A750	non-LRE	L858R	Overall
**Gefitinib**	17/28 (60.7%)	0/3	7/23 (30.4%)	24/54 (44.4%)
**Erlotinib**	4/9 (44.4%)	0/1	0/5 (0%)	4/15 (26.7%)
**Afatinib**	4/6 (66.7%)	0/0	1/5 (20.0%)	5/11 (45.5%)
**Overall**	19/31 (61.3%)	0/3	8/31 (25.8%)	27/65 (41.5%)

### Computational modeling results

After the attribute ranking analysis, we found that the probability of acquiring T790M after taking first-line TKIs (gefitinib, erlotinib and afatinib) was highly correlated with the patient’s initial EGFR mutation type. Patients with the delE746_A750 mutation have much higher probability of acquiring T790M than patients with the L858R mutation or exon 19 non-LRE deletions. Moreover, delE746_A750 and L858R accounted for about 92% of all patients we studied and delS752_I759 was the most common exon 19 non-LRE deletions^24^. In order to investigate this relationship further, we carried out molecular modeling and analysis for these three EGFR mutations, delE746_A750, delS752_I759 and L858R. We explored the motion pattern of each mutant-TKI complex in MD simulations.

Although the EGFR L858R-gefitinib complex (2ITZ) is available in the Protein Data Bank (PDB)^25^, no information of other EGFR mutant and drug complexes exist in the public domain. Furthermore, the EGFR kinase domain should contain residues from 696 to 1022, but 2ITZ was not completely recorded. Only three segments, 697–865, 876–990 and 1002–1020, are available in 2ITZ. In this regard, we first needed computational modeling to predict the 3D structures of EGFR mutant-TKI complexes for the three EGFR mutations and the three TKIs (gefitinib, erlotinib and afatinib).

### Complete wild-type (WT) EGFR structure

**Figure 1:**
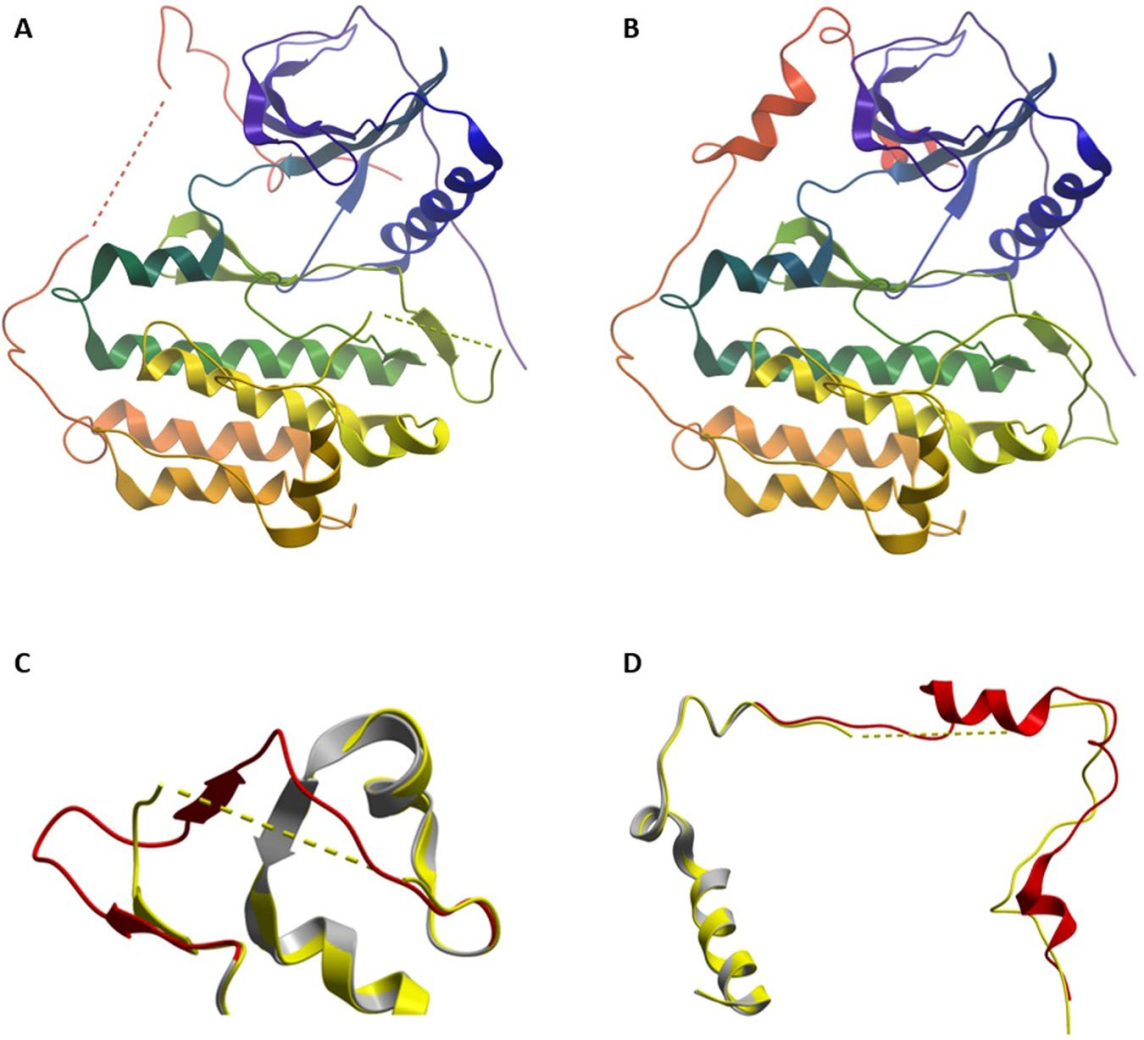
Computational modeling results for the complete WT EGFR structure. (**A**) The initial structure 2ITY. (**B**) The generated complete WT EGFR structure. (**C**) The segments of 3IKA (residues 858–879). (**D**) The segments of 3W2S (residues 987–1019). In (**C**,**D**), the yellow part (2ITY) and the grey part (3IKA or 3W2S) aligned very well. The red segments replaced the corresponding yellow segments to generate the complete WT EGFR structure.

For the first step, we generated the complete WT EGFR structure using structure alignment methods. The results were shown in Fig. 1. 2ITY was used as the initial structure and segments of 3IKA (residues 858–879) and 3W2S (residues 987–1019) were used as the complements to the lost parts of 2ITY.

### EGFR mutant-TKI complex

**Figure 2:**
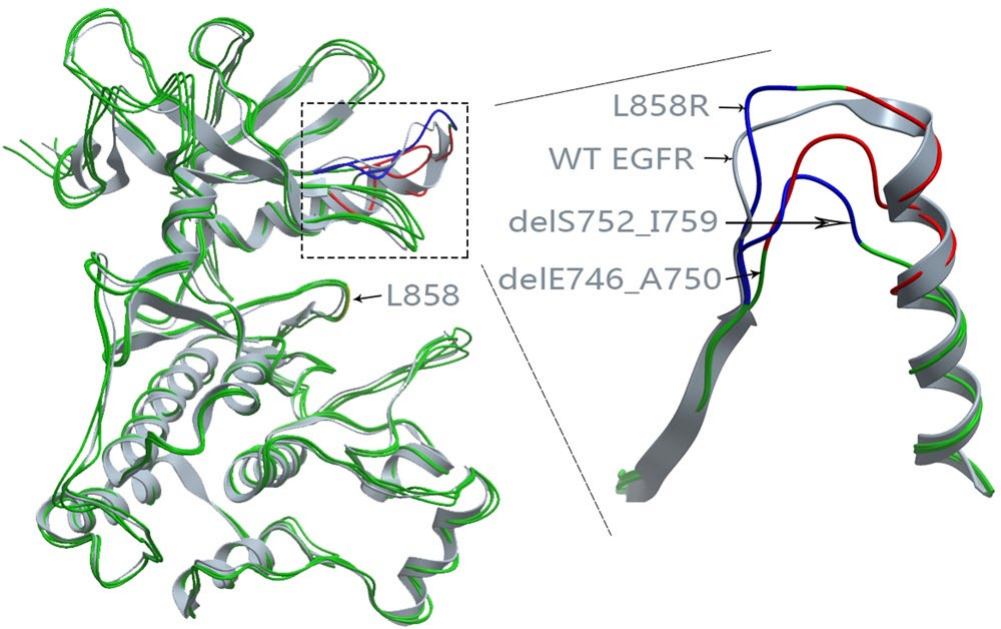
Computational modeling results for L858R, delE746_A750 and delS752_I759. (**A**) Overall structures for these three mutants. (**B**) Details in the deletion sites. Blue parts are the sites of E746 to A750 and red parts are the sites of S752 to I759.

After obtaining the complete WT EGFR structure, we generated the structures for all EGFR mutants, (delE746_A750, L858R and delS752_I759) using Rosetta. Their results are shown in Fig. 2A. We realized that the structures of the three EGFR mutants (green) looked very similar to that of WT EGFR (gray). When examined more closely, however, some differences could be discerned in the deletion sites among these three different mutational types, as shown in Fig. 2B. Compared with the WT EGFR structure, the deletion sites of the deletion mutants were rearranged. Little difference was observed in the mutational site of L858R for the backbone.

**Figure 3:**
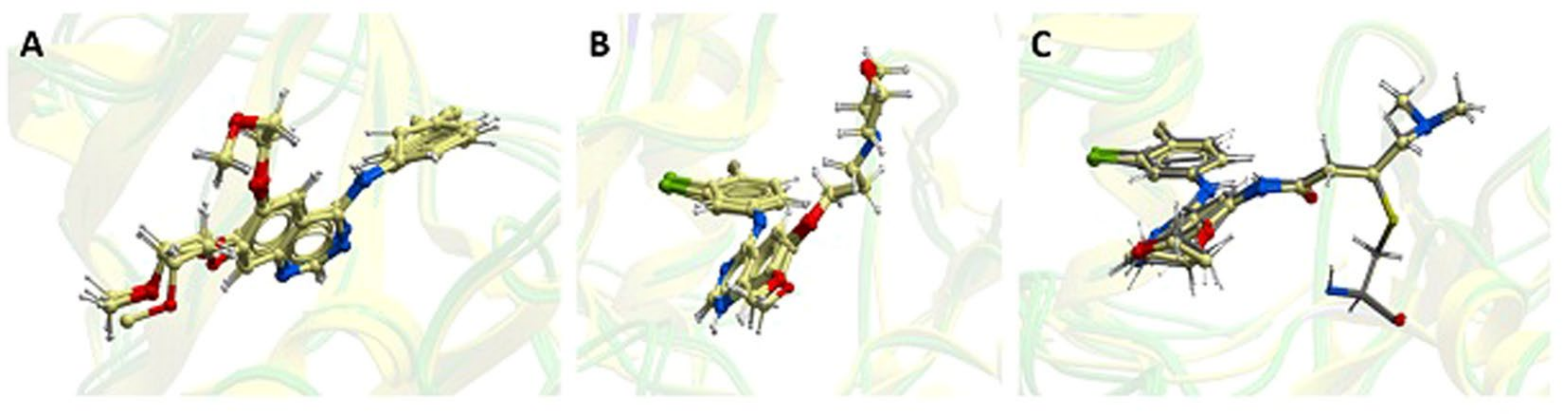
The results of the aligned TKIs. (**A**) Gefitinib. 2ITY was the template. (**B**) Erlotinib. 1M17 was the template. (**C**) Afatinib. 4G5J was the template. Each figure contained four TKIs. Three of them were for the EGFR mutant-TKI complexes and the other one was for the template.

Then we aligned the above EGFR mutants to templates of the three TKIs and placed the TKIs to proper positions to generate EGFR mutant-TKI complexes. 2ITY, 1M17 and 4G5J were selected as the templates for EGFR mutant-gefitinib, erlotinib and afatinib complexes. The results were displayed in Fig. 3. Each diagram contained the same four TKIs. Three of them are for the EGFR mutant-TKI complexes and the other one was for the template. Afatinib was covalently bound to EGFR kinase domain and we combined afatinib and EGFR C797 into a new block “AFA”, as shown in Fig. 3C. The diagrams showed these TKIs were aligned well with RGFR mutants although there were minor differences produced in the energy minimization procedure.

### Quality assessment of generated structures

Quality assessment of the predicted models is an important part in protein structure prediction. In this study, we used three commonly used methods to assess the quality of our predicted EGFR mutant-TKI complexes. The first one is QMEAN Z-score^26, 27^, which estimates the quality of a single protein structure by relating it to a representative set of high resolution experiment structures, with a higher Z-score corresponding to a better quality of the structure. The second method is Verify3D^28^, which assesses the compatibility of a model with its amino acid sequence based on its location and environment. A value bigger than 80% will pass the verification. We also used Ramachandran Plot to validate the structures^29^. These three assessment methods were applied to the nine EGFR mutant-TKIs complexes and the reference structure 2ITY. The results were shown in Table 4. We confirmed that, compared to the reference structure 2ITY, all 9 EGFR mutant-TKIs complexes passed the quality assessment with satisfactory results.

**Table 4:** Quality assessment of the nine EGFR mutant-TKIs complexes and the reference structure 2ITY.

	Z-score	Verify3D	Ramachandran Plot Favored/Allowed/Outlier
2ITY	−1.38	88.00%	85.4%/10.5%/4.1%
delE746_A750-gefitinib	−1.87	90.97%	93.7%/4.7%/1.6%
delS752_I759-gefitinib	−1.90	89.90%	93.0%/6.1%/1.0%
L858R-gefitinib	−1.63	92.70%	93.8%/5.9%/0.3%
delE746_A750-erlotinib	−1.41	90.65%	91.8%/7.0%/1.3%
delS752_I759-erlotinib	−2.01	88.60%	93.3%/5.8%/1.0%
L858R-erlotinib	−1.54	92.38%	93.1%/6.5%/0.3%
delE746_A750-afatinib	−1.65	88.35%	93.0%/5.4%/1.6%
delS752_I759-afatinib	−2.11	88.89%	95.8%/2.9%/1.3%
L858R-afatinib	−1.97	92.70%	93.8%/5.9%/0.3%

EGFR: epidermal growth factor receptor.

### Molecular dynamics (MD) simulations and residue stability results

In order to investigate the relationship between patients’ initial EGFR mutation types and the presence of T790M as the second mutation after taking gefitinib, erlotinib or afatinib, we analyzed the stability from the motion patterns of residues around the residue T790 for each mutant-TKI complex. Through performing MD simulations (simulation time 10 ns) we obtained a trajectory of 5000 frames for each EGFR mutant-TKI complex.

**Figure 4:**
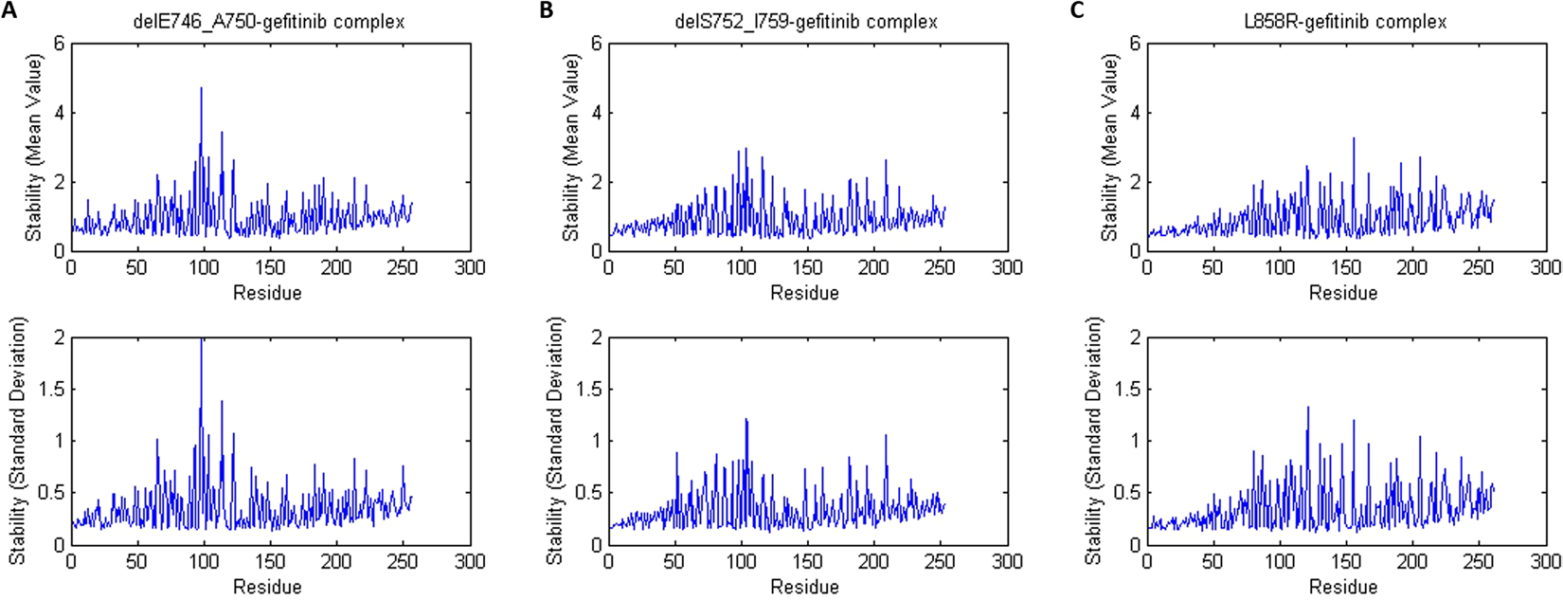
The residue stability of (**A**) delE746_A750-gefitinib complex, (**B**) delS752_I759-gefitinib complex and (**C**) L858R-gefitinib complex. The upper panels showed the mean value and the lower panels showed the standard deviation.

We calculated the stability of each residue in each EGFR mutant-TKI complex. Figure 4 showed the residue stability of delE746_A750-gefitinib complex, delS752_I759-gefitinib complex and L858R-gefitinib complex, respectively. Only residues from 716 to 976 were presented since there might be a degree of arbitrariness of the stability of the head (residue 697–715) and the tail (residue 977–1019) of each EGFR mutant. The head and the tail were roughly selected because the first helix started at about residue 716 and the last helix ended at about residue 976. In Fig. 4, the horizontal axes were the residue indices and we rearranged their order according to their distance to the residue T790. Residue index 0 corresponded to the residue T790 itself and residue index 1 corresponded to the closest residue to T790. From Fig. 4, we noticed that the stability, in terms of both the mean value and the standard deviation, of the residues in the EGFR mutant-gefitinib complexes around T790 was relatively higher than residues far from T790. For EGFR mutant-erlotinib and EGFR mutant-afatinib complexes, the results were similar.

**Figure 5:**
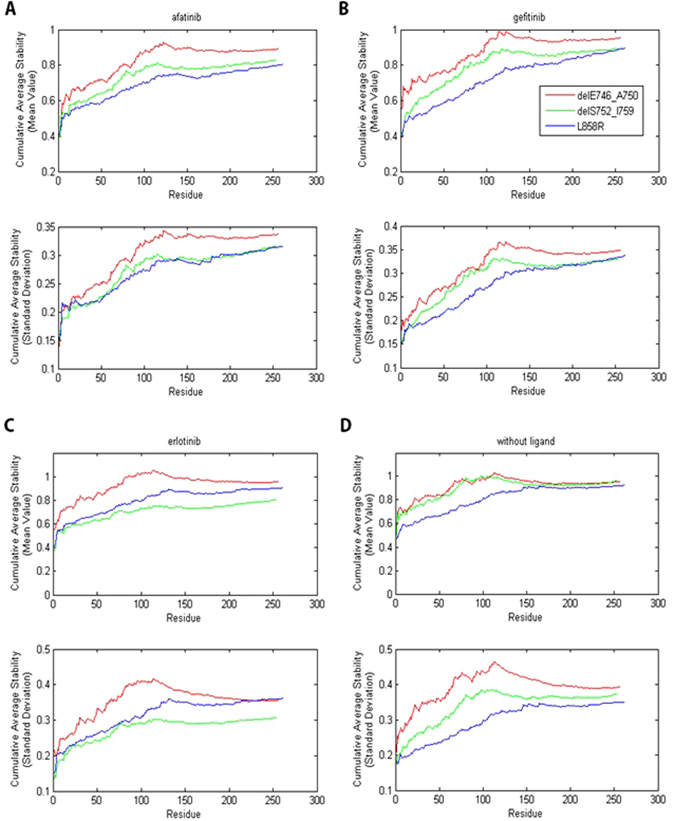
The cumulative average stability for (**A**) EGFR mutant-afatinib complexes, (**B**) EGFR mutant-erlotinib complexes, (**C**) EGFR mutant-gefitinib complexes and (**D**) EGFR mutants without ligand. The cumulative average stability for residue index k corresponded to the average stability of the first k residues closest to the residue T790.

To compare the residue stability around T790 for different EGFR mutants-TKI complexes, we calculated the cumulative average for all stability results. Specifically, the cumulative average stability for residue index k in Fig. 5 corresponded to the average stability of the first k residues closest to T790. The cumulative average stability, especially the first dozens of values, reflected the overall residue stability around T790. From Fig. 5, we found that for all three TKIs, delE746_A750 had a higher value (i.e. lower stability) around T790 than the other two mutations, delS752_I759 and L858R. This result was consistent with the fact that patients with delE746_A750 were more likely to acquire a second T790M mutation than patients with delS752_I759 or L858R after taking gefitinib, erlotinib or afatinib as 1^st^ line therapy. On the other hand, for afatinib and gefitinib, delS752_I759 has lower stability around T790 than L858R while delS752_I759 has higher stability around T790 than L858R for erlotinib. Because the probability of acquiring T790M mutation for patients with delS752_I759 or L858R was low, and there were only three patients whose tumor harbored delS752_I759 mutation, it was thus difficult to compare the residue stability of these two mutations. We also carried out simulations for EGFR mutants without ligand (Fig. 5D). We found that in this case, the stability results also roughly the same as those discussed above. We also observed that combining with a ligand could increase the stability of the EGFR mutant (see Supplementary Fig. S1).

## Discussion

Patients with metastatic NSCLC which harbored activating EGFR mutations (exon 19 deletions and exon 21 L858R mutation) respond very well to first generation TKI (gefitinib and erlotinib) and second generation TKI (afatinib) as first-line treatment, as exemplified by international phase III RCT^8–19^. However, more than 50% of such patients will develop acquired resistance to these TKIs because of a second mutation T790M^20^. Exploring the mechanisms of acquiring T790M after failure to TKIs is crucial to subsequent decision making and study design in future preclinical and clinical drug trials.

Plasma biopsy by ddPCR is one of the most sensitive techniques and platforms of detecting acquired T790M mutation after EGFR TKI therapy^30, 31^. The DNA templates by ddPCR were separated into thousands or even millions of individual parallel PCR reactions. The detection of the signal after amplification by droplet flow cytometry indicates the presence (positive) or absence (negative) of the target sequence. It allows for detection of mutant cfDNA in a high background of wild-type cfDNA, leading to a high sensitivity. The use of ddPCR is one of the strengths in our study that gives an accurate presentation of the genomic landscape of the tumor microenvironment, as compared to single tumor biopsy which only offers the genomic picture of a particular tumor focus.

In this study, we found that bone metastasis, initial EGFR mutational type and the number of sites of metastases correlated well with development of acquired T790M mutation after taking first-line TKI with or without subsequent TKI or systemic chemotherapy. If patients had both bone metastasis and delE746_A750 mutation, the probability of acquiring T790M was even higher. On the other hand, 61.3% of patients with the delE746_A750 mutation acquired T790M, while the percentages for exon 19 non-LRE deletions and L858R were 0 and 25.81%, respectively. It seemed that patients with delE746_A750 would have a higher probability of acquiring T790M than those with exon 19 non-LRE deletions mutation, though a definite conclusion could not be made owing to the small number of patients with exon 19 non-LRE deletions.

Based on our results that patients who had delE746_A750 mutation were more likely to acquire T790M after targeted treatment than those with exon 19 non-LRE deletions or L858R mutation, we conducted computational studies by the use of molecular dynamics. Specifically, we first generated the structures for all EGFR mutant-TKI complexes involved using computational modeling methods. Through MD simulations, we obtained a trajectory of 5000 frames for each complex. Then we calculated the stability of each residue in each EGFR mutant-TKI complex. We found that the stability of residues in the EGFR mutant-gefitinib complexes around T790 was relatively higher than residues far from T790 for all three EGFR mutations and the results were similar when the other two EGFR-mutant-TKI complexes we studied. We also found that for all three TKIs, delE746_A750 has lower stability around the residue T790 than the other two mutations namely delS752_I759 and L858R. These results echoed with our clinical observation that patients with delE746_A750 were more likely to acquire a second T790M mutation than patients with delS752_I759 or L858R after taking gefitinib, erlotinib or afatinib. However, it is noteworthy that T790M mutation is no longer prognosticative of a worse survival^32–34^, especially when T790M specific TKIs have been developed and evaluated showing excellent objective responses and survival outcomes^35, 36^. In particular, osimertinib has been approved by the United States, Japan and Korea for metastatic NSCLC with acquired T790M mutation after prior failure to first or second generation TKI^35^. To the best of our knowledge, our study is the first providing a plausible computational explanation why progressive disease develops in those with T790M mutation after the first and second TKIs. The findings here can lead us to a better understanding of mechanism of acquiring the T790M mutation after targeted treatment and will be beneficial to EGFR-mutated NSCLC treatment design.

Our study also has some limitations. First of all, only 4 patients had paired tumor re-biopsies with plasma re-biopsies for acquired T790M mutation. Though ddPCR is one of the most sensitive methods of plasma biopsy for T790M mutation, the concordance rate between plasma and tumor re-biopsies was around 60–70%^37, 38^. Nevertheless, plasma re-biopsies provide a broader representation of the genomic landscape of all subpopulations of tumors within a patient. Secondly, we created the structures of EGFR mutant-erlotinib and EGFR mutant-afatinib for this study specially because there was no previous literature showing their molecular structures. However, we believe that our methods of computational modeling are credible as they are based on standard protocols. In addition, quality assessment of our EGFR mutant-TKI complexes has been performed using three methods to ensure their stability. Thirdly, we could not provide a clear answer if our study results can be extrapolated to other rarer subtypes of sensitizing EGFR mutations, including delL747_P753insS and other insertion/substitutions subtypes in exon 19 and L861Q in exon 21 as we reported previously^24^. A larger cohort with more dedicated methods of sequencing e.g. next-generation sequencing at baseline before TKI therapy for these rarer mutational subtypes is warranted to investigate their correlation with T790M development.

In conclusion, this is the first study demonstrating the predictive factors for acquired T790M mutation and the instability of binding between exon 19 delE746_A750 and TKIs leading to higher rates of acquired T790M development which correlated with the clinical observations in our patients. Our results have provided some insight on future directions of research investigating the mechanisms of acquired T790M mutation, which is essential to the development of novel mutation-specific TKIs.

## Methods

### Patient eligibility and data collection

Prior approval by local institutional review board (Institutional Review Board of University of Hong Kong/Hospital Authority Hong Kong West Cluster) was obtained before study commencement. The study protocol and the experiment methods in this study were also approved by the same institutional review board. All patients provided written informed consent before recruitment. All clinical investigations and management were conducted according to the principles of Declaration of Helsinki. Patients were eligible for this study if they were histologically or cytologically diagnosed to have metastatic NSCLC, if they had known activating EGFR mutations (either exon 19 deletions or exon 21 L858R mutation), if they had clinical benefits (as defined by Jackman criteria) from first or second-generation EGFR-TKI (either gefitinib, erlotinib or afatinib) as first-line therapy and if they had confirmed radiologically confirmed PD, as defined by the Response Evaluation Criteria in Solid Tumors (RECIST) version 1.1, while still receiving such EGFR-TKI^39, 40^. Regular imaging surveillance at baseline and then every 3 to 4 months after first-line TKI with or without subsequent systemic therapy with either computed tomography (CT) of the brain, chest and abdomen or position emission tomography with integrated CT scan (PET-CT) was performed for tumor response assessment. Those who had pre-treatment exon 20 T790M mutation were excluded from this study. The number of lines of prior TKI therapies and/or systemic chemotherapy ± anti-VEGF therapy with bevacizumab was not limited in this study. After progressive disease (PD) to first-line TKI therapy with or without subsequent TKI therapies or systemic therapies, blood taking for plasma biopsy for cell-free tumor DNA (cfDNA) by ddPCR and/or tissue re-biopsy followed by Sanger sequencing for detecting acquired T790M mutation was performed from all patients within 2 weeks after radiological confirmation of PD to their last line of systemic therapy. The method and the platform for ddPCR was previously described^41, 42^. In brief, ddPCR assay was conducted by droplets generation using QX200 generator (Bio- Rad Laboratories, Inc., Hercules, CA, USA), followed by endpoint PCR reactions using C1000 (Bio-Rad) and droplet flow cytometry readings using QX200 reader (Bio- Rad). Analyzed data were processed using QuantaSoft (Bio-Rad) software. Several mutations including all types of exon 19 deletions, exon 21 L858R mutation and exon 20 T790M mutation could be detected simultaneously.

### Attribute ranking

To evaluate the importance of each attribute contributing to the presence of T790M mutation in plasma or tumor biopsy after TKI therapy, attribute ranking was performed as follows. We selected 24 attributes that were assessable and complete for all patients (Table 5), including patients’ baseline characteristics and their treatment modalities. We further sub-categorized age into different subgroups: age ≥55, 60, 65, 70, 75 and 80 years. For the initial EGFR mutational types, we divided them into three groups: exon 19 LRE deletions, exon 19 non-LRE deletions and L858R point mutation. Weka 3.8.0^43, 44^ was used to perform attribute ranking. We used multiple attribute evaluators available in Weka, including Chi-squared Ranking Filter, Correlation Ranking Filter, Filtered Attribute Evaluator, Information Gain Ranking Filter, ReliefF Ranking Filter and Symmetrical Uncertainty Ranking Filter for the subsequent analysis. For all these six methods, we used Ranker as the search method, which ranked attributes by their individual evaluations. Default parameters were applied to all attribute evaluators and the search method. The results were compared after performing attribute ranking with these six methods.

**Table 5:** Attributes selected for attribute ranking.

	Attribute	Description
1	Age_55	Age ≥55 years
2	Age_60	Age ≥60 years
3	Age_65	Age ≥ 65 years
4	Age_70	Age ≥70 years
5	Age_75	Age ≥75 years
6	Age_80	Age ≥80 years
7	Sex	Sex
8	Smoking	Smoking history at baseline
9	Lung_met	Lung metastasis at baseline
10	Pleural_met	Pleural metastasis at baseline
11	Brain_met	Brain metastasis at baseline
12	Liver_met	Liver metastasis at baseline
13	Bone_met	Bone metastasis at baseline
14	Distant_LN_met	Distant lymph node metastasis at baseline
15	Number_sites_met	Number of sites of metastases at baseline
16	Initial_EGFR_mutation	Type of initial EGFR mutation before TKI therapy
17	Number_lines_TKI	Number of lines of TKI used before liquid and/or tumor re-biopsy
18	Number_lines_chemo	Number of lines of chemotherapy before liquid and/or tumor re-biopsy
19	First_TKI	Use of first TKI
20	Second_TKI_used	Use of second TKI
21	Third_TKI_used	Use of third TKI
22	First_chemo_used	Use of first-line chemotherapy
23	Second_chemo_used	Use of second-line chemotherapy
24	Third_chemo_used	Use of third-line chemotherapy

EGFR: epidermal growth factor receptor, LN: lymph node.

### EGFR mutant-TKI complex modeling

Our method for EGFR mutant-TKI complex modeling consisted of three main steps. The first step was to generate a complete structure of the WT EGFR kinase domain. In our study, Molsoft ICM-Browser (http://www.molsoft.com/icm_browser.html)^45^ was employed and we used the visualization and alignment functions of this tool to complete this task. Specifically, we first selected three EGFR structures, 2ITY, 3W2S and 3IKA, from the PDB and we aligned them together using Molsoft ICM-Brower. We used 2ITY (EGFR WT-gefitinib complex) as the initial structure and segments of 3IKA (EGFR T790M-WZ4002 complex) and 3W2S (EGFR WT-compound4 complex) were used as the complements to the lost parts of 2ITY. Combining these three structures we were able to produce a complete structure of EGFR kinase domain. Then, we performed an energy minimization on the structure using Amber^46^ to optimize the structure.

The second step was to generate structures for all EGFR mutants involved with the complete WT EGFR structure with the method previously described by us^47^. Specifically, Rosetta^48^ ddg_monomer protocol was used to generate the EGFR point mutation and Rosetta comparative modeling (CM) protocol was used to generate the mutations of amino acids deletion. For the point mutation, Rosetta first replaced the side-chain of the residue at the mutation position and then optimize the rotamers of all residues using its side-chain optimization module. For mutations of amino acids deletion, Rosetta first aligned these mutant sequences to the template WT EGFR sequence and then built the well-aligned regions using the CM protocol. Next, loop modeling with the fragment library was applied to rebuild the missing parts. After the final refinement step we were able to generate the required structure.

The third step was to combine the above EGFR mutants with the three TKIs to generate EGFR mutant-TKI complexes. The basic method was to align the EGFR mutant to a template and then add the TKI of the template to the current EGFR mutant. Specifically, we chose 2ITY as the template for EGFR mutant-gefitinib complexes, 1M17 for EGFR mutant-erlotinib complexes and 4G5J for EGFR mutant-afatinib complexes. Since afatinib is an irreversible TKI which is covalently bound to EGFR kinase domain, we manually created a covalent bond between afatinib and the EGFR mutant to produce the EGFR mutant-afatinib complexes. Here, we deleted the H atom of the thiol side chain in the cysteine residue at position 797 of exon 20. However, this structure cannot be used directly for molecular dynamics simulations in Amber. We needed to combine afatinib and EGFR C797 into a new block, which will be called “AFA”. Only in this way, Amber could deal with EGFR-afatinib as a single connected object, and not two separate unconnected objects like EGFR-gefitinib or EGFR-erlotinib complexes. Finally, we performed an energy minimization on the structure using Amber to optimize these structures.

### Molecular dynamics (MD) simulations

We performed MD simulations using Amber12. We first employed the antechamber program^49^ to assign atomic charges and atom types for gefitinib, erlotinib and “AFA”. Then, the first step of MD simulations was preparation of the coordinate (.inpcrd) and topology (.prmtop) files using the LEaP tool in Amber. To achieve this, we first loaded the Amber force fields ff12SB and gaff to construct the molecular topologies. Then we loaded the EGFR mutant and combined it with the corresponding TKI to create a complex. Subsequently, we created a solvent environment for each system with the TIP3P water model. The truncated octahedral water box was used and a 10-angstrom buffer was set around the solute in each direction. After neutralizing the solvated system, the coordinate and topology files were saved for further processing.

The next step was minimization and equilibration of the system to guarantee a stable simulation. This step was the same with the procedures applied before^50^. Specifically, we first performed a 1000-step energy minimization on the system to remove bad contacts within the solute. Then we heated the system for 50 ps from 0 K to 300 K. Lastly, we implemented a density equilibration for 50 ps and a constant-pressure equilibration for 500 ps on the system. For minimization, heating and density equilibration, we applied a weak restraint with a weight of 2 (in kcal/mol-Å^2) on all non-H atoms of the solute. We validated the equilibration of the system by observing the stability of the temperature, density, energy and root mean square deviation (RMSD) of the system (see Supplementary Fig. S2). Once the system was equilibrated, the key production MD simulations of 10 ns were performed with constant temperature and constant pressure. Trajectory frames were sampled every 2 ps, resulting in 5000 frames for each EGFR mutant-TKI complex.

### Residue stability calculation

After obtaining the trajectory data of each EGFR mutant-TKI complex, we evaluated the stability of each residue of the complex. The major method was to calculate the difference between each frame and a reference frame. For the reference frame, there were several choices, including the original structure (the structure of the EGFR mutant-TKI complex from modeling and 3D structure prediction), one of the frames of the trajectory, or the average structure of the trajectory. In this study, we used the average structure as the reference frame because it was more representative of all the frames in the trajectory. However, prior to calculating the average structure, it was required to remove the translation and rotation of each frame. The cpptraj program in AmberTools12 was used and the position of each frame was best-fit to the original structure of the EGFR mutant-TKI complex. Furthermore, we considered only non-hydrogen atoms since hydrogen atoms were too small and too light when compared to heavy atoms.

To calculate the residue stability, we first defined distance (A1, A2) (distance between two atoms A1 and A2) as the Euclidean distance between points A1(x1, y1, z1) and A2(x2, y2, z2) (Equation (1)). We then defined the distance between each residue in each frame and the corresponding residue in the reference frame as residue_dist_i,j_, equal to the average of the distances between corresponding atoms in this residue (Equation (2)). The residue stability represented the fluctuation of each residue in all trajectory frames around their average position. It consisted of two parts. One was the average distance between each residue in each frame and the corresponding residue in the reference structure (Equation (3)) and the other part was the standard deviation of the distances (Equation (4)). Both of them could reflect the stability of each residue.

1 $${\rm{distance}}({{\rm{A}}}_{1},{{\rm{A}}}_{2})=\sqrt{{({{\rm{x}}}_{1}-{{\rm{x}}}_{2})}^{2}+{({{\rm{y}}}_{1}-{{\rm{y}}}_{2})}^{2}+{({{\rm{z}}}_{1}-{{\rm{z}}}_{2})}^{2}}$$

2 $${\rm{residue}}\_{{\rm{dist}}}_{i,j}=\frac{{\sum }_{k}distance({{\rm{A}}}_{{\rm{i}},{\rm{j}},{\rm{k}}},{{\rm{ref}}}_{i,k})}{K}$$

3 $${\rm{stability}}\_{{\rm{mean}}}_{i}=\frac{{\sum }_{j}{\rm{residue}}\_{{\rm{dist}}}_{i,j}}{J}$$

4 $${\rm{stability}}\_{{\rm{std}}}_{i}=\sqrt{\frac{{\sum }_{j}{(residue\_dis{t}_{i,j}-stability\_mea{n}_{i})}^{2}}{J-1}}$$

where A_i,j,k_ stands for the coordinates of the k^th^ atom in the i^th^ residue of the j^th^ frame, ref_i,k_ represents the coordinates of the k^th^ atom in the i^th^ residue of the reference structure, residue_dist_i,j_ corresponds to the distance between the i^th^ residue of the j^th^ frame and the i^th^ residue of the reference structure, stability_mean_i_ and stability_std_i_ represent the stability of the i^th^ residue, K is the number of non-hydrogen atoms in the corresponding residue, and J is the number of frames in the trajectory.

To investigate the residue stability around the residue T790, we evaluated the average distance between each residue and the residue T790. Specifically, we first calculated the center coordinates of each residue in each frame (Equation (5)). We defined the distance between two residues as the distance between their centers. Then the average distance between each residue and the residue T790 was computed for all position frames (Equation (6)). We studied the stability of each residue as well as their distance to T790 to investigate the residue stability around the residue T790 and compare the residue stability of different EGFR mutant-TKI complexes.

5 $${\rm{residue}}\_{{\rm{center}}}_{i,j}=\frac{{\sum }_{k}{{\rm{A}}}_{{\rm{i}},{\rm{j}},{\rm{k}}}}{K}$$

6 $$dist\_to\_t{790}_{i}=\frac{{\sum }_{j}distance({{\rm{center}}}_{i,j},{{\rm{center}}}_{T790,j})}{J}$$

where residue_center_i,j_ stands for the center coordinates of the i^th^ residue in the j^th^ frame, and dist_to_T790_i_ is the distance between the i^th^ residue and the residue T790.

### Statistical analysis

Fisher’s exact tests were performed for each high-ranking attribute for the statistical significance for the presence of acquired T790M mutation. Statistical significance was defined as p < 0.05 (two-sided). All statistical analyses were performed using GraphPad InStat version 3.10 for Windows, GraphPad Software, San Diego California USA, www.graphpad.com.

## Electronic supplementary material

Supplementary Information
