# Supplementary material for: Deciphering mechanisms of acquired T790M mutation after EGFR inhibitors for NSCLC by computational simulations
Source: Sci Rep. 2017 Jul 26;7:6595. doi: 10.1038/s41598-017-06632-y (PMC5529360; doi:10.1038/s41598-017-06632-y)
Supplement: Supplementary file 1 — Supplementary Information [file 41598_2017_6632_MOESM1_ESM.pdf]

SUPPLEMENTARY INFORMATION

Deciphering mechanisms of acquired T790M mutation after EGFR inhibitors for NSCLC by computational simulations

Bin Zou, Victor H. F. Lee, Lijiang Chen, Lichun Ma, Debby D. Wang and Hong Yan

SUPPLEMENTARY FIGURES:

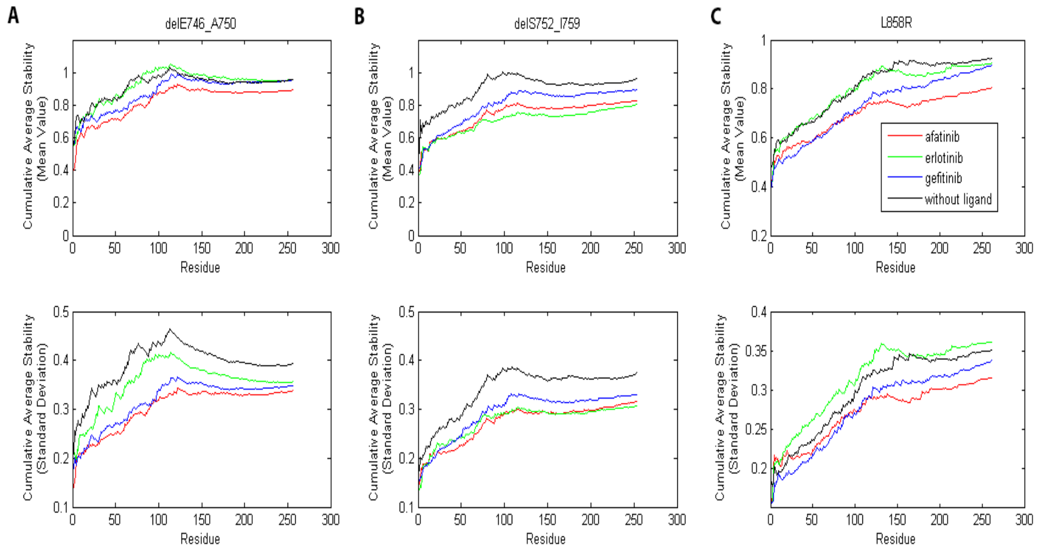

Supplementary Fig. S1. Comparison of the cumulative average stability between EGFR mutant-TKI complexes and EGFR mutants without ligand. The cumulative average stability for residue index k corresponded to the average stability of the first k residues closest to the residue T790. It was shown that, EGFR mutants without ligand (black) had a lower stability (larger values in the figures) than EGFR mutant-TKI complexes in most cases. This implied that combination with a ligand could increase the stability of the EGFR mutant. One exception was that the stability (standard deviation) of the L858R-erlotinib complex was lower than that of L858R. The reason might be that the stability of L858R was very high relative to delE746_A750 and delS752_I759 (Figure 4) and in this case the effect of the ligand (erlotinib) might not always work for L858R. It was also observed that EGFR mutant-afatinib complexes (red) demonstrated the highest stability in most cases. This is because that afatinib covalently binds to EGFR mutants, which could make EGFR mutant-TKI complexes more stable.

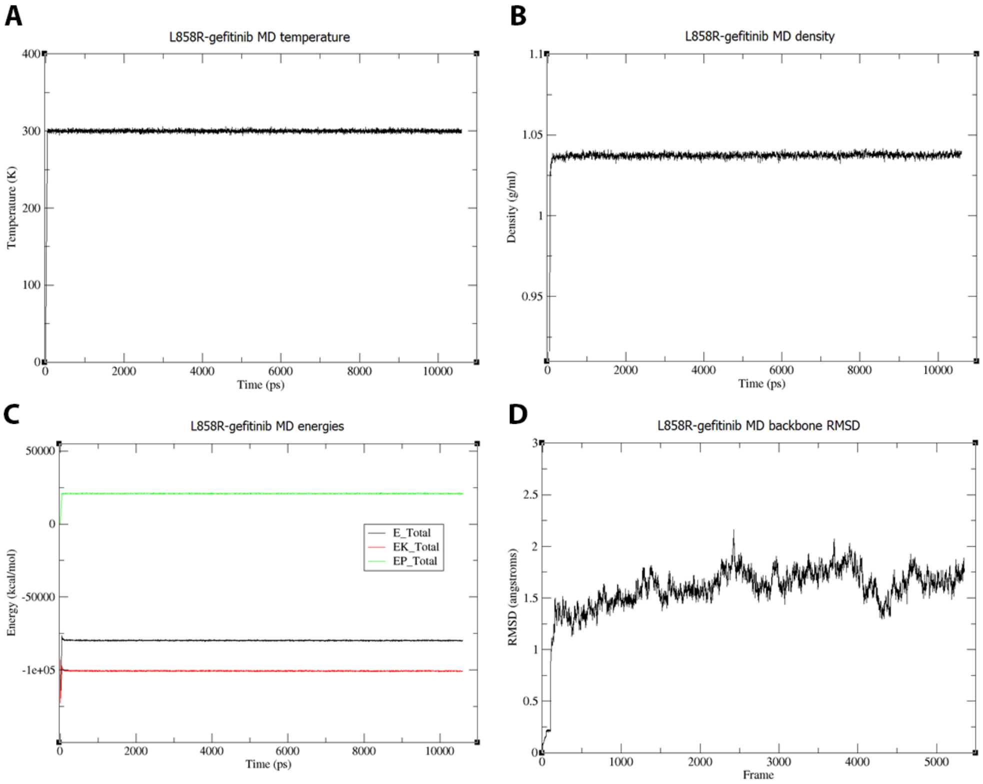

Supplementary Fig. S2. The (A) temperature, (B) density, (C) energy and (D) backbone RMSD of the L858R-gefitinib complex as functions of time. It was observed that the system finally achieved a stable state after a series of equilibration operations.
